# Free-Living Bacteria May Utilize Chromosomal Toxin–Antitoxin Systems to Mediate K Sensing and Control by Continuously Modulating the Ratio of Injury: Repair Throughout the Life Cycle

**DOI:** 10.3390/toxins18040183

**Published:** 2026-04-12

**Authors:** Stephen J. Knabel, Aubrey Mendonca

**Affiliations:** 1 Department of Food Science, The Pennsylvania State University, University Park, PA 16802, USA; 2 Department of Food Science and Human Nutrition, Iowa State University, Ames, IA 50011, USA; amendon@iastate.edu; 3 Interdepartmental Microbiology Program, Iowa State University, Ames, IA 50011, USA

**Keywords:** toxin–antitoxins, injury, repair, ratio, K sensing and control

## Abstract

A recent publication proposed that the main biological function of chromosomal toxin–antitoxin systems (TASs) in free-living bacteria is to optimize fitness by mediating K Sensing and Control via a Nutrient-Responsive Cybernetic System. Viable cell density data were consistent with analog (continuous) regulation of population dynamics and cellular physiology throughout the life cycle; however, exactly how bacteria utilize TASs to regulate this was not explained in that publication. Two different concepts of injury have been proposed in the field of microbiology: (1) injury due to external physical and chemical stresses, which lead to sublethal (reversible) or lethal (irreversible) injury depending on the degree of injury, and (2) injury due to internal, self-inflicted stresses mediated by TA toxins. While self-inflicted injury due to TA toxins has been recognized as playing a role in growth arrest and dormancy, which can be reversed by repair, there is little support for TA toxins causing irreversible programmed cell death under normal physiological conditions. The purpose of the present paper was to explain how merging the above two concepts of injury might reveal how TASs optimize the fitness of free-living bacteria under normal physiological conditions by continuously regulating the ratio of injury: repair throughout the life cycle.

## 1. Introduction

A recent publication [1] proposed that the main biological function of chromosomal toxin–antitoxin systems (TASs) in free-living bacteria is to optimize biological fitness by mediating K (carrying capacity) Sensing and Control (KSC) throughout the life cycle via a Nutrient-Responsive Cybernetic System (NRCS). In that NRCS model, the intracellular concentration of nutrients ‘feeds back’ to control the concentration of free TA toxins, which regulate population dynamics and cellular physiology. Viable cell density data were consistent with chromosomal TA toxins modulating population dynamics and cellular physiology in an analog (continuous) manner throughout the life cycle. There were two exceptions to this continuous variation: (1) in the log phase, excess intracellular nutrients result in the synthesis of excess antitoxins relative to toxins [2], which neutralizes all free toxins, thus enabling rapid growth of all cells, and (2) in the long-term survival (LTS) phase, nutrients are in equilibrium with viable cell density at K, which results in the presence of a moderate but constant intracellular concentration of free toxins, which holds all surviving cells in growth and death arrest (dormancy) [1]. However, exactly how free-living bacteria may utilize chromosomal TASs to achieve this was not clearly explained in that previous publication. Therefore, the purpose of the present paper is to present a mechanism by which free-living bacteria may utilize chromosomal TASs to efficiently regulate population dynamics and cellular physiology in an analog (continuous) manner throughout the life cycle.

## 2. The Biological Function of Chromosomal TA Systems in Free-Living Bacteria

**Figure 1:**
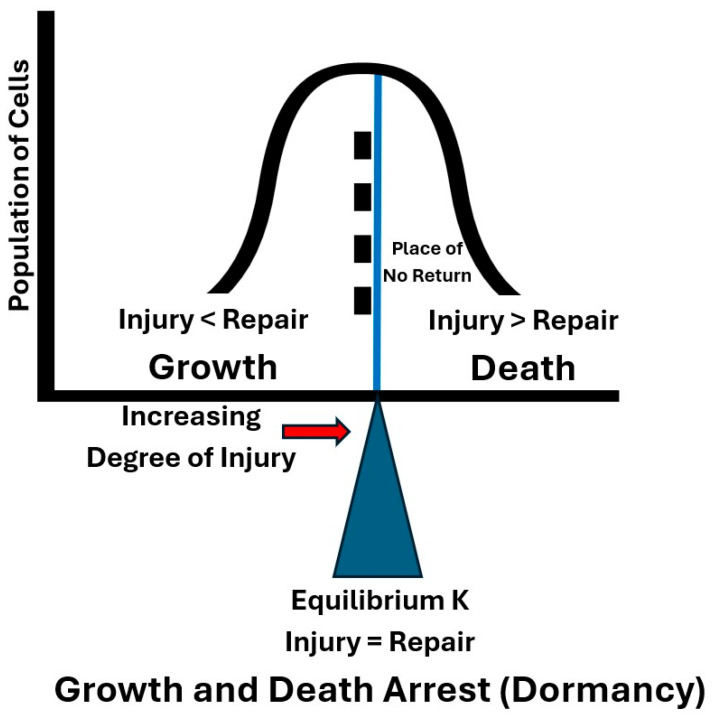
Hypothetical heterogeneous distribution of injury caused by decreasing intracellular nutrients and subsequent release of free TA toxins in a population of bacterial cells that have overshot the carrying capacity (K). Where Injury < Repair, those cells grow until they reach equilibrium K; where Injury = Repair at K, those cells are in Growth and Death Arrest (Dormancy); and where Injury > Repair above K, those cells have entered the Place of No Return (Death).

Understanding metabolism is key to understanding life and involves both the catabolism (degradation) and anabolism (synthesis) of complex macromolecules [3]. Many TA toxins degrade or modify biomolecules that are critical to various cellular processes in free-living bacteria [4]. TA toxins are typically enzymes that are thought to interfere with [4], affect [5], modulate [6], inhibit [7], disrupt [8], damage [9], or harm [10] various essential cellular processes inside free-living bacteria, resulting in growth arrest [7,8,10,11] or cell death [9,12,13]. However, TA toxins have not been described in scientific literature as causing ‘injury’. Perhaps this is due to scientists reserving the concept of injury for those harmful effects caused by external stresses and not recognizing that injury could also be a beneficial phenomenon caused by internal stresses that bacteria inflict upon themselves via TA toxins. Numerous researchers failed to observe phenotypic effects of chromosomal TA toxins in wild-type cells under normal physiological conditions and thus resorted to ectopic (artificial) overexpression of chromosomal TA toxins to observe reversible growth arrest and the formation of dormant persister cells [6,7,9,11,12,14,15] or programmed cell death (PCD) [9,12]. Most TA toxins inhibit translation, whereas other processes (DNA synthesis, cell wall synthesis, and membrane integrity) are targeted by only a few other toxin–antitoxin families [4]. In contrast to their role in growth arrest (dormancy) and persistence [5,6,7,8,11,14,15], the proposed role of chromosomal TA toxins in PCD [9,12,13] remains highly debated and less widely accepted. To our knowledge, only one publication [1] has linked chromosomal TA toxins to density-dependent PCD under normal physiological conditions. However, reversible and irreversible injuries in bacteria due to multi-hit injury kinetics have long been widely accepted concepts in the fields of food and environmental microbiology, where injury is caused by external stresses like heating, freezing, drying, starvation, irradiation, high pressure, and various chemicals [16,17,18,19,20,21,22,23]. In contrast, the concept of injury has rarely been linked to self-inflicted damage caused by chromosomal TA toxins in wild-type, free-living bacteria under normal physiological conditions. However, Amitai et al. [9] reported that damage due to extended ectopic overexpression of the chromosomal TA toxin MazF caused cells of *E. coli* to reach an irreversible ‘point of no return’. This ‘point of no return’ is consistent with the concept of bacterial death, which is defined as the point where the extent of injury is beyond the ability of a cell to repair injury and resume growth [24]. We accept this definition of bacterial death. Knabel et al. [1] proposed that TA toxins would cause PCD in free-living bacteria under normal physiological conditions when the population density of viable cells exceeds the carrying capacity (K). In our model, this results in the degradation of existing antitoxins and the lack of synthesis of new antitoxins, and the subsequent dissociation (release) of high levels of free toxins from preformed toxin–antitoxin complexes [1]. Such injury and death are consistent with the field of food microbiology, where heating, freezing, drying, and gamma-radiation have been shown to cause increasing degrees of injury with increasing intensity and time of exposure to these processes [17,25]. The degree of injury in a population of bacterial cells is heterogeneous due to the probabilistic, multi-hit nature of injury, which explains the first-order death kinetics associated with the above processes [26]. Under this scenario, sublethal (reversible) injury occurs in those cells in the population that receive a lower number of hits, while lethal (irreversible) injury occurs in those cells in the population that receive such a high number of hits that the degree of injury exceeds the ability of the cell to repair and subsequently reproduce (Figure 1) [24]. Equation (1) (below) is a generalized formula for calculating the degree of injury in individual bacterial cells by stresses such as heating, freezing, drying, and irradiation. While the concept of injury is well established in the fields of food and environmental microbiology, it has yet to be proven that TA-toxin-mediated injury explains growth arrest or programmed cell death under normal physiological conditions. Further experiments are needed to test this hypothesis.


Degree of Injury = Intensity of Stress (Hits/Unit Time) × Time = Total Number of Hits
(1)

Given the above equation, the degree of injury in a cell can be enhanced by increasing (1) the intensity of stress (hits per unit time) and/or (2) time. When the total number of hits exceeds some critical limit, injury exceeds the ability to repair and death of the cell occurs (Figure 1) [24,26]. Thermal pasteurization is a good example of this concept applied to populations of bacterial cells, where the same degree of irreversible injury (log reductions or death) of a population of foodborne pathogens can be achieved by either High-Temperature Short-Time (HTST) or Low-Temperature Long-Time (LTLT) pasteurization processes. In the case of TA toxins, the intensity of stress (Hits/Unit Time) is determined by the intracellular concentration of free toxin. According to our model, when the viable population density goes above K the degree of injury due to free TA toxins in some cells exceeds their ability to repair injury (Injury > Repair) and death ensues (Figure 1). As previously mentioned, this is consistent with the findings of Amitai et al. [9] using ectopic overexpression of the chromosomal TA toxin MazF.

## 3. Injury and Repair Are Inversely Proportional and Tied to Intracellular Nutrient Concentration

**Figure 2:**
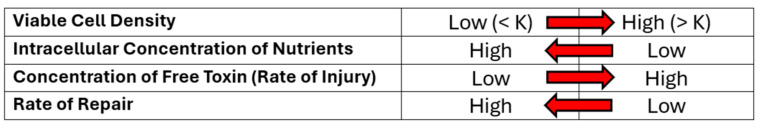
Fate of bacterial cells throughout the life cycle depends on the intracellular concentration of nutrients, which determines the rates of injury and repair, which are inversely proportional to one another. Red arrows indicate the direction of the hypothetical continuous variation of each parameter from low to high.

**Figure 3:**
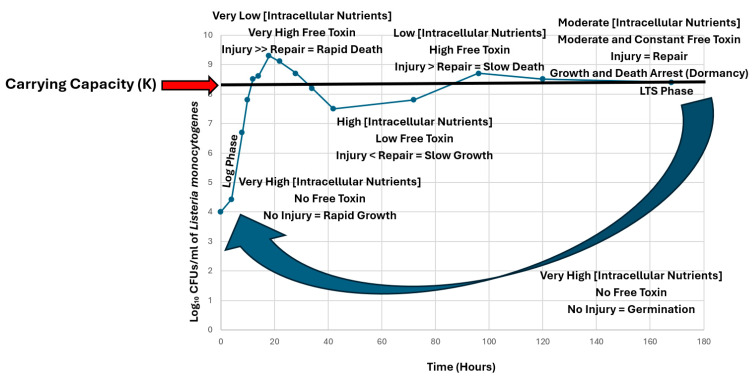
Model of how chromosomal toxin–antitoxin systems may modulate population dynamics and cellular physiology in a continuous manner throughout the life cycle via the ratio of injury–repair. Below K Injury < Repair, which results in growth; above K Injury > Repair, which results in death; at K Injury = Repair, which results in growth/death arrest (dormancy). When nutrients are again in excess, dormant cells germinate and grow (large blue curved arrow). Cells of *Listeria monocytogenes* were grown in trypticase soy yeast extract broth (TSBYE) at 35 °C. Aliquots were sampled and plated on TSBYE Agar, which was incubated at 35 °C for 48 h prior to counting colonies and calculating Log_10_ Colony Forming Units (CFUs) per ml (blue dots and lines). The figure was modified from Figure 2 in reference [1].

In our previous NRCS model [1], intracellular nutrient concentration feeds back to control the concentration of antitoxin and thus the intracellular concentration of free toxin, which controls various emergent properties throughout the life cycle of free-living bacteria. We predict that there is a continuum in the ratio of injury–repair throughout the life cycle, and both are tied to viable cell density and thus intracellular nutrient concentration (Figure 2). According to our model, when the viable cell density is below K, intracellular nutrient concentration is high and thus antitoxins are synthesized in excess [2], which neutralizes the activity of all TA toxins, allowing rapid growth (Figure 3). However, when the viable population density exceeds K, intracellular nutrient concentration is low and thus antitoxins can no longer be synthesized at a high level, which results in the release of high concentrations of free TA toxins and thus a high level of degradation/disruption (injury) of various vital cellular biomolecules, which results in rapid death (Figure 3). In summary, the NRCS model predicts that at low viable cell density (high intracellular nutrient concentration), the degree of injury to vital macromolecules is low and synthesis (repair) is high, while at high viable cell density (low intracellular nutrient concentration), the reverse is true (Figure 2). In the field of food microbiology, it is well known that sublethal injury can be repaired most rapidly by incubating cells in nutrient-rich, non-selective media at favorable temperatures [18]. In the case of injury due to TA toxins, we propose that injury is repaired faster with increasing intracellular nutrient concentration below K (Figure 2). This is consistent with the results of Amitai et al. [9] who reported that cells in which MazF was ectopically overexpressed reached a ‘point of no return’ (death) sooner in a minimal medium than in a nutrient-rich medium.

## 4. Chromosomally Encoded TA Toxins May Optimize the Fitness of Free-Living Bacteria Throughout the Life Cycle by Causing Self-Inflicted Injury

The authors recognize the long-standing debate as to whether TA toxins cause programmed cell death (PCD) ***or*** growth arrest. The Engelberg-Kulka group published several papers which suggested that the TA toxin MazF regulated death and survival at high viable cell density. Kolodkin-gal et al. [27] argued that PCD was mediated by a quorum-sensing extracellular death factor (EDF). Amitai et al. [28] hypothesized that MazF did not directly kill bacterial cells or inhibit their growth but resulted in the selective synthesis of death and survival proteins. Wen et al. [29] demonstrated that cells of *Listeria monocytogenes* died rapidly in ‘spent’ trypticase soy yeast extract broth (TSBYE) when inoculated at cell densities greater than K, but reproduced and did not die when inoculated at viable cell densities below K. In both cases, the final viable cell densities tapered off slowly to eventually reach K [29]. However, when cells were inoculated at low levels into fresh ‘nutrient-rich’ TSBYE, cells grew rapidly and overshot K and then damped down to K [29]. In our previous publication [1], we proposed an NRCS model in which TA toxins cause **both** PCD above K ***and*** growth arrest at K **depending on the intracellular concentration of free toxin**, **which is regulated by the intracellular nutrient concentration**. In that model, both PCD above K and growth/death arrest (dormancy/persistence) at K enhance the biological fitness of free-living bacteria by preserving nutrients required for their long-term survival. In the present paper, we hypothesize that the intracellular nutrient concentration and thus the concentration of free toxin ultimately determine the ratio of injury–repair, which determines whether PCD or growth arrest occurs (Figure 3). Hutchinson [30] realized that rapidly growing biological populations cannot act instantaneously and thus they first overshoot K and then undergo a damped oscillation before reaching K. To accommodate this type of delay-driven damped oscillation, Hutchinson incorporated a time lag term (τ) in the logistic equation to create a delay logistic equation. This type of damped oscillation above and below K is what we observed in the model presented in the present paper (Figure 3). We hypothesize that as rapidly reproducing populations overshoot K, there is a delay in death (injury > repair) due to a delay in antitoxin degradation and release of free toxin. We also hypothesize that after the population drops below K, there is a delay in reproduction (injury < repair) due to a delay in the synthesis of new antitoxin and the sequestration of free toxin. We further hypothesize that the damped oscillation in viable cell density finally reaches equilibrium K in the LTS phase, where the concentration of free toxin is moderate and constant and thus injury = repair (Figure 3). Experiments are needed to test whether any of the above hypotheses are valid and might explain how TA toxins regulate both PCD and growth/death arrest (dormancy/persistence) during normal physiological conditions.

The fields of food and environmental microbiology have long recognized that injury caused by various external stresses can result in sublethal (reversible) injury or lethal (irreversible) injury, depending on the degree of injury. However, these fields have not recognized that injury could also be due to internal self-inflicted stresses mediated by TA toxins. In contrast, the field of toxin–antitoxins has long recognized that TA toxins cause self-inflicted damage to various essential cellular macromolecules in free-living bacteria; however, it has not recognized the role of TA toxins in irreversible PCD in wild-type free-living bacterial cells under normal physiological conditions. While external stresses like heat affect many different macromolecules within the cell and thus produce a broad range of diverse types of injury, TA toxins being enzymes catalyze very specific types of injury by targeting specific sites in specific macromolecules [4]. For example, the endoribonuclease TA toxin MazF in *E. coli* only cleaves RNA molecules at specific ACA sites [31]; if no ACA sites are present in a particular transcript, no cleavage occurs. Such selective targeting of RNA by MazF may enable cleavage (injury) of those transcripts that result in growth and death arrest (dormancy), while causing no cleavage (injury) to transcripts that code for proteins needed for long-term survival. In addition to rapid and specific injury being mediated by enzymes (TA toxins), repair of that injury is also mediated by enzymes (i.e., RNA polymerase and ribosomes) that catalyze the specific synthesis of new macromolecules to replace those that have been injured. Thus, enzyme-catalyzed, self-inflicted injury mediated by chromosomal TASs and enzyme-catalyzed, self-mediated repair would enable rapid and efficient phenotypic transitions in free-living bacteria.

The present paper proposes to merge the above two concepts of injury: (1) reversible and irreversible injury and (2) self-inflicted, enzyme-catalyzed injury caused by chromosomal TA toxins. With this merger, rapid, specific, self-inflicted, internal injury due to chromosomal TA toxins may cause both (1) reversible injury at K, which results in growth/death arrest (dormancy) and (2) irreversible injury above K, which results in PCD (Figure 3). In this new view of injury, while TA toxins cause *harm to individual bacterial cells*, they more importantly provide a *benefit to the population of cells* by continually varying the ratio of injury: repair throughout the life cycle. This optimizes fitness by efficiently regulating population dynamics and cellular physiology in concert with intracellular nutrient concentration, resulting in KSC via an NRCS [1]. This view is consistent with free-living bacteria being multicellular organisms, which benefit by the actions of TA toxins on individual cells [1]. Like sublethal injury due to heating and other food processes, the NRCS model (1) predicts that sublethal injury due to TA toxins can be reversed when the intracellular nutrient concentration is high below K, resulting in (1) the synthesis of antitoxins which neutralize TA toxins and (2) the synthesis of new essential macromolecules to replace those that have been damaged by free toxins. At K the concentration of viable cells is in equilibrium with intracellular nutrient concentration and thus the rate of injury equals the rate of repair, which produces a moderate but constant concentration of free toxin, which results in growth and death arrest (dormancy) (Figure 3) [1]. The TA toxin MazF is thought to cause reversible dormancy by changing the translational program and specifically affecting several pathways: co-translational quality control, ribosome hibernation and recycling, cell division, and cell wall thickness [15]. In contrast to reversible injury, the NRCS model [1] predicts that lethal (irreversible) injury due to TA toxins occurs above K where intracellular nutrients are limited and thus the degree of injury exceeds the ability of the cell to repair injury (Figure 1 and Figure 3) [9]. While this may sound counterintuitive [32], death of cells above K is an altruistic phenomenon predicted by the theory of inclusive fitness [33,34]. In that theory, death of some members of the population (altruists) enhances the reproductive success (fitness) of the remaining viable, but dormant, cells (recipients) by *preserving* nutrients critical for their long-term survival (Figure 3) [1]. In summary, free-living bacteria may utilize chromosomal TA toxins to continually modulate the ratio of injury–repair throughout the life cycle via self-inflicted, multi-hit injury kinetics, which optimizes fitness by efficiently regulating growth below K, PCD above K, growth and death arrest (dormancy) at K, and subsequent germination and growth below K (Figure 3).

## 5. Conclusions

Chromosomal TASs may cause the ratio of injury–repair to vary continuously with intracellular nutrient concentration throughout the life cycle. This may cause the smooth/continuous transition between the different emergent phenotypes of growth, death, growth and death arrest, and germination (Figure 3) [1]. This continually varying ratio of injury–repair may enable TASs to optimize fitness throughout the life cycle of free-living bacteria by efficiently mediating KSC via an NRCS [1].

## 6. Future Directions

For endoribonuclease TA toxins like MazF, the changing ratio of injury–repair could be validated by measuring the degree of RNA cleavage (injury) and degree of new RNA synthesis (repair) throughout the life cycle. Application of chromosomal TA toxinmediated injury might be applied to the development of novel antibiotics. One strategy for accomplishing this is via the use of antisense RNAs [35] targeted to degrade RNAs that encode antitoxins, which would result in the release of high concentrations of free TA toxins that could inhibit or kill bacterial pathogens. A suitable candidate for this approach would be the TAS *mazEF*, as it is common in the genomes of free-living bacteria and free MazF toxin has been associated with causing PCD [9,12,13]. In this scenario, RNA encoding the antitoxin MazE would be targeted for degradation by antisense RNA, which would result in the release of elevated levels of free MazF toxin. This could result in bacterial inhibition or death depending on the level of free toxin released. The ratio of injury–repair could also be artificially altered using antisense RNAs designed to degrade RNAs required for repair of injury. The above approaches might lead to the development of novel antisense RNA antibiotics for controlling numerous free-living bacterial pathogens, including those that are currently resistant to many conventional antibiotics [35].

## Data Availability

No new data were created or analyzed in this study.
